# An integrated analysis identified mitochondrial ribosomal protein MRPL3 as a potential prognostic biomarker and therapeutic target in pancreatic cancer

**DOI:** 10.7150/jca.114067

**Published:** 2025-07-11

**Authors:** Wuhan Yang, Huiyan Deng, Teng Pan, Xiaokun Zhang, Li Peng, Shubin Wang

**Affiliations:** 1 Department of Hepatobiliary Surgery, The Fourth Hospital of Hebei Medical University, Shijiazhuang, Hebei 050011, P.R. China.; 2 Department of Pathology, The Fourth Hospital of Hebei Medical University, Shijiazhuang, Hebei 050011, P.R. China.; 3 Department of Oncology, Shijiazhuang First Hospital, Shijiazhuang, Hebei 050011, P.R. China.; 4 Department of General Medicine, The Fourth Hospital of Hebei Medical University, Shijiazhuang, Hebei 050000, P.R. China.

**Keywords:** pancreatic cancer, mitochondria, MRPL3, prognostic biomarkers, immune microenvironment.

## Abstract

Mitochondria play a crucial role in tumor metabolism. Mitochondrial ribosomal protein L3 (MRPL3) is a core component of the mitochondrial ribosome. However, its role in pancreatic cancer (PC) remains unclear. We investigated the biological functions and underlying mechanisms of MRPL3 in PC. The expression of MRPL3 was analyzed using public databases. Prognostic significance was evaluated using Kaplan-Meier survival analysis and univariate/multivariate Cox regression. Functional enrichment analysis was performed to identify MRPL3-associated signaling pathways. In addition, immune cell infiltration and tumor mutational burden (TMB) analyses were conducted to explore the relationship between MRPL3 expression and the tumor microenvironment. Tumor immune dysfunction and exclusion (TIDE) scores and drug sensitivity analyses were used to assess the therapeutic implications of MRPL3. Western blotting and immunohistochemistry (IHC) were performed to validate MRPL3 expression and evaluate their prognostic significance in clinical PC samples. *In vitro* experiments were performed to determine the effects of MRPL3 silencing on PC cell behavior. MRPL3 expression was notably increased in PC and associated with an unfavorable prognosis in public cohorts. Functional enrichment and immune infiltration analyses revealed that high MRPL3 expression was associated with damage to the G2/M DNA checkpoint, increased Th2 cell infiltration, and reduced natural killer (NK) cell activity. Furthermore, high MRPL3 expression corresponded to lower immunotherapy sensitivity and higher chemotherapy sensitivity. The IHC analysis confirmed that high MRPL3 expression is associated with significantly shorter overall survival in PC (hazard ratio [HR] = 2.13, 95% confidence interval [CI] = 1.35-3.34, *p* = 0.001). *In vitro* experiments demonstrated that MRPL3 knockout significantly suppressed PC proliferation, migration, and invasion. MRPL3 promotes PC progression, immune evasion, and therapeutic resistance, contributing to an unfavorable prognosis. It may serve as a promising biomarker and potential target for individualized treatment strategies.

## Introduction

Pancreatic cancer (PC) is an aggressive malignancy characterized by nonspecific early symptoms and is typically diagnosed at advanced stages, leading to an overall 5-year survival rate of less than 10% 1,2. Pancreatic ductal adenocarcinoma (PDAC) accounts for more than 90% of all PCs; the term “pancreatic cancer” is generally used to refer to PDAC 2. Radical surgical resection is the only curative treatment available. However, the highly invasive nature of PC often results in early recurrence, limited drug efficacy, and poor prognosis 3. Therefore, it is crucial to explore the mechanisms underlying tumor progression. Identifying novel therapeutic targets to improve PC treatment outcomes and precise biomarkers for better prognostic stratification are urgently needed.

Mitochondria contribute to cellular respiration and energy metabolism, primarily through ATP production via oxidative phosphorylation 4. In cancer, mitochondria support rapid tumor cell proliferation through metabolic reprogramming and ATP generation 5,6. Moreover, mitochondrial dysfunction, resulting from mutations in mitochondrial genes, electron transport chain defects, or increased oxidative stress, can promote tumorigenesis 7. In addition, mitochondria modulate cellular metabolic states and signaling pathways, thereby influencing the tumor microenvironment (TME) and tumor progression 8. Consequently, mitochondria are promising therapeutic targets, and investigation of their functions could provide novel insights and strategies for cancer treatment.

Mitochondrial ribosomal protein L3 (MRPL3) is a component of the 39S mitochondrial ribosomal subunit, which is ubiquitously expressed in eukaryotic cells 9,10. Its primary functions include the facilitation of mitochondrial protein translation and the regulation of oxidative phosphorylation 11. Previous bioinformatics studies have indicated that MRPL3 is highly expressed and potentially prognostic in several cancers, including breast and liver cancers; however, these findings lack experimental validation, and the role of MRPL3 in PC remains unknown 9,12.

In this study, we used bioinformatic analyses to examine MRPL3 expression and its correlation with clinical prognosis in PC. We validated the prognostic value of MRPL3 using immunohistochemistry (IHC) in our cohort of patients with PC. Subsequently, we developed an MRPL3-based nomogram for precise prognostic stratification. Finally, we performed *MRPL3* knockdown experiments in PC cell lines to elucidate its effects on tumor cell proliferation, migration, and invasion. Our findings suggest that MRPL3 is a valuable target, potentially providing personalized treatment strategies and improving the prognostic management of PC.

## Materials and Methods

### Data Acquisition and Differential Expression Analysis

RNA sequencing data and clinical information for PC and adjacent normal tissues were obtained from The Cancer Genome Atlas (TCGA) (https://tcga-data.nci.nih.gov/tcga/) and Genotype-Tissue Expression (GTEx) (gtexportal.org/home/) databases. The RNA-seq data were normalized to transcripts per million (TPM) for pan-cancer differential expression analysis. RNA-seq data were obtained from the GEO dataset GSE183795.

### Analysis of Differentially Expressed Genes (DEGs)

Patients with PC were divided into two groups based on high and low MRPL3 expression levels, defined by the median MRPL3 levels. DEGs between groups were identified using the “DESeq2” package, applying significance thresholds of |log₂ fold change (FC)| > 2 and adjusted *p* < 0.05. The results were visualized using a volcano plot that highlighted the five most significantly upregulated or downregulated genes.

### Enrichment Analysis

The “clusterProfiler” package was used to conduct functional enrichment analyses of the DEGs, including the Gene Ontology (GO) and Kyoto Encyclopedia of Genes and Genomes (KEGG) pathways. Gene set enrichment analysis (GSEA) utilized the “c2.cp.all.v2022.1. Hs” dataset. The top five positively and negatively enriched pathways (ranked by adjusted *p-*values) were visualized.

### Immune Infiltration Analysis

Immune infiltration was evaluated using CIBERSORTx to quantify 22 immune cell subsets in the PC samples 13. The Wilcoxon test was employed to compare high and low MRPL3 groups, whereas the Spearman correlation assessed the relationship between MRPL3 expression and immune infiltration levels. Visualizations were generated using the “ggClusterNet” package.

### Protein-Protein Interaction (PPI) Analysis

Cytoscape software (version 3.9.0) was used to construct a protein-protein interaction (PPI) network. Hub genes were identified by intersecting the top 15 genes ranked by seven cytoHubba algorithms (MCC, DMNC, MNC, Degree, EPC, Closeness, and EcCentricity) using the “UpSet” package. Correlations between MRPL3 and hub genes were visualized using heat maps.

### Tumor mutation burden (TMB) analysis

*MRPL3* mutations and copy number variations were explored using the cBioPortal (https://www.cbioportal.org/). Somatic genomic alterations, including single-nucleotide polymorphisms (SNPs), insertions/deletions, TMB, and mutation frequencies, were analyzed using the “maftools” package. The top 20 most frequently mutated genes were illustrated 14.

### Single-Cell Sequencing Analysis

Single-cell RNA-seq data from GSE154778 (8,000 cells from 10 primary PC tumors and 6,926 cells from 6 metastatic tumors) were analyzed using the “MAESTRO” and “Seurat” packages. The cells were re-clustered using the t-SNE method.

### Tumor Stemness Score Analysis

Tumor stemness was evaluated using RNA-based stemness scores to determine its association with MRPL3 expression.

### MRPL3 Expression in PC Cell Lines

MRPL3 mRNA expression data from 40 PC cell lines were obtained from the Cancer Cell Line Encyclopedia (CCLE) database (https://sites.broadinstitute.org/ccle), and the expression was visualized using bar graphs.

### Drug Sensitivity and Immunotherapy Response Evaluation

Half-maximal inhibitory concentration (IC_50_) values of PC-related chemotherapeutics were retrieved from the Genomics of Drug Sensitivity in Cancer (GDSC) database. Associations between MRPL3 expression and drug sensitivity were analyzed using the “oncoPredict” and “pRRophetic” packages. Immunotherapy responses were evaluated using the Tumor immune dysfunction and exclusion (TIDE) platform, and immune checkpoint gene expression was compared between the two groups.

### Identification of Potential Therapeutic Compounds

A connectivity map (CMap) database was used to identify potential therapeutic compounds. The top 30 upregulated and 30 downregulated DEGs (ranked based on log_₂_|FC|, and *p*-adjust < 0.05) were input into the L1000 platform to predict candidate compounds. Five compounds with the lowest enrichment scores were selected as potential MRPL3-related therapeutic candidates.

### Clinical Sample Collection

This retrospective analysis included patients with PC who underwent curative resection at the Fourth Hospital of Hebei Medical University between January 2016 and December 2021. The inclusion criteria were as follows: (1) pathological diagnosis of PDAC, (2) no previous antitumor therapy before surgery, and (3) undergoing curative surgical resection. The exclusion criteria were as follows: (1) patients with concurrent malignant tumors and (2) incomplete clinical and pathological data or loss to follow-up. A total of 142 patients were included in this study. Paraffin-embedded tumor specimens and the corresponding clinical data, including sex, age, postoperative chemotherapy, and complications, were collected. Postoperative complications such as bleeding, infection, and pancreatic fistula were defined as those requiring invasive interventions and extended hospitalization. Follow-up was continued until December 31, 2024. Ethical approval was granted by the hospital's ethics committee (no. 2023KS182), and written informed consent was obtained from all participants.

### Cell Culture

The human PC cell line SW1990 was procured from the Chinese Academy of Sciences (Shanghai, China) and cultured in Dulbecco's modified Eagle medium (DMEM) supplemented with 10% fetal bovine serum (FBS) and 1% penicillin-streptomycin at 37°C in a 5% CO₂ humidified incubator.

### Cell Transfection

SW1990 cells were transfected with MRPL3-specific shRNA (GeneChem, Shanghai) using Lipofectamine 3000 (Invitrogen). The sequences for sh-MRPL3 are as follows: sh-MRPL3 1:5'-CCUUUAGAGUUGGUCUUAUTT-3', sh-MRPL3 2:5'-GCUACAUCCAUAUUGGAAUTT-3'. *MRPL3* knockdown efficiency was assessed by western blotting at 48 h post-transfection.

### Western Blotting

Protein extraction was followed by sodium dodecyl sulfate acrylamide-polyacrylamide gel electrophoresis and transfer to polyvinylidene fluoride (PVDF) membranes, which were subsequently incubated overnight with primary antibodies (MRPL3 and tubulin; dilution, 1:1000), followed by incubation with a secondary antibody. The bands were visualized using enhanced chemiluminescence and quantified by comparing the grayscale intensity with internal controls.

### IHC

Paraffin-embedded PC tissue sections and normal tissues were processed for IHC staining using a primary anti-MRPL3 antibody (Proteintech; Wuhan; Dilution 1:200). Staining was scored independently by two pathologists based on the intensity and percentage of positive cells. The intensity was scored on a scale of 1 (weak), 2 (moderate), or 3 (strong), whereas the percentage of positive cells was categorized as 1 (0-5%), 2 (6-25%), 3 (26-50%), or 4 (> 50%). The final IHC score was calculated by multiplying the two scores, with a high MRPL3 expression defined as a score ≥ 8 15.

### Survival and Cox Regression Analyses

Kaplan-Meier survival curves were used to compare the overall survival (OS) between the high and low MRPL3 expression groups. Univariate and multivariate Cox regression analyses were performed to evaluate prognostic factors, with variables showing *p* < 0.1 in the univariate analysis included in the multivariate analysis. Statistical significance was set at *p* < 0.05.

### LASSO Regression

The “glmnet” package was utilized to conduct the least absolute shrinkage and selection operator (LASSO) regression with 10-fold cross-validation. This study aimed to identify the optimal prognostic features for developing a prognostic model.

### Prognostic Nomogram Construction and Validation

A nomogram was constructed using the “rms” package to predict 1-, 3-, and 5-year OS. Model calibration and predictive accuracy were evaluated using calibration plots and time-dependent receiver operating characteristic (ROC) curves.

### CCK-8 Assay

PC cells (1,000 cells per well) were seeded in 96-well plates. The CCK-8 reagent (Dojindo, Japan) was added to each well and incubated for 2 h at 37°C. The absorbance at 450 nm was measured to evaluate cell viability, and each experiment was conducted in triplicate.

### Colony Formation Assay

PC cells (800 cells/well) were plated in six-well plates and incubated for 14 days. The resulting colonies were fixed in 4% paraformaldehyde, stained with 1% crystal violet, and counted under a microscope.

### Wound Healing Assay

SW1990 cells were grown to confluence in six-well plates. A sterile pipette tip was used to create a wound in the cell monolayer, and the detached cells were removed by washing with phosphate-buffered saline. Cell migration to the wounded area was observed at 0 and 48 h. The wound area was measured using the ImageJ software, and the wound closure rate was calculated to quantify cell migration.

### Transwell Assay

Cell migration and invasion were assessed in 24-well transwell chambers (Corning; NY, USA). Matrigel was used to pre-coat the upper chamber for the invasion assays, which was filled with serum-free medium, and the lower chamber contained a medium with 10% fetal bovine serum (FBS) as a chemoattractant. The cells on the upper membrane were carefully removed after 24 h of incubation. The cells that traversed the lower surface of the membrane were fixed, stained with 1% crystal violet, and counted microscopically.

### Statistical Analysis

Statistical analyses were conducted using GraphPad Prism 9.0 and R 4.1.3. Data are presented as mean ± standard deviation (SD). RNA-seq data were normalized to transcripts per million (TPM) and log_2_-transformed. Comparisons between groups were performed using Student's *t*-test or one-way analysis of variance (assuming equal variances), with statistical significance noted as *p < 0.05, **p < 0.01, and ***p < 0.001.

## Results

### High MRPL3 Expression in PC Based on Public Databases

**Figure 1:**
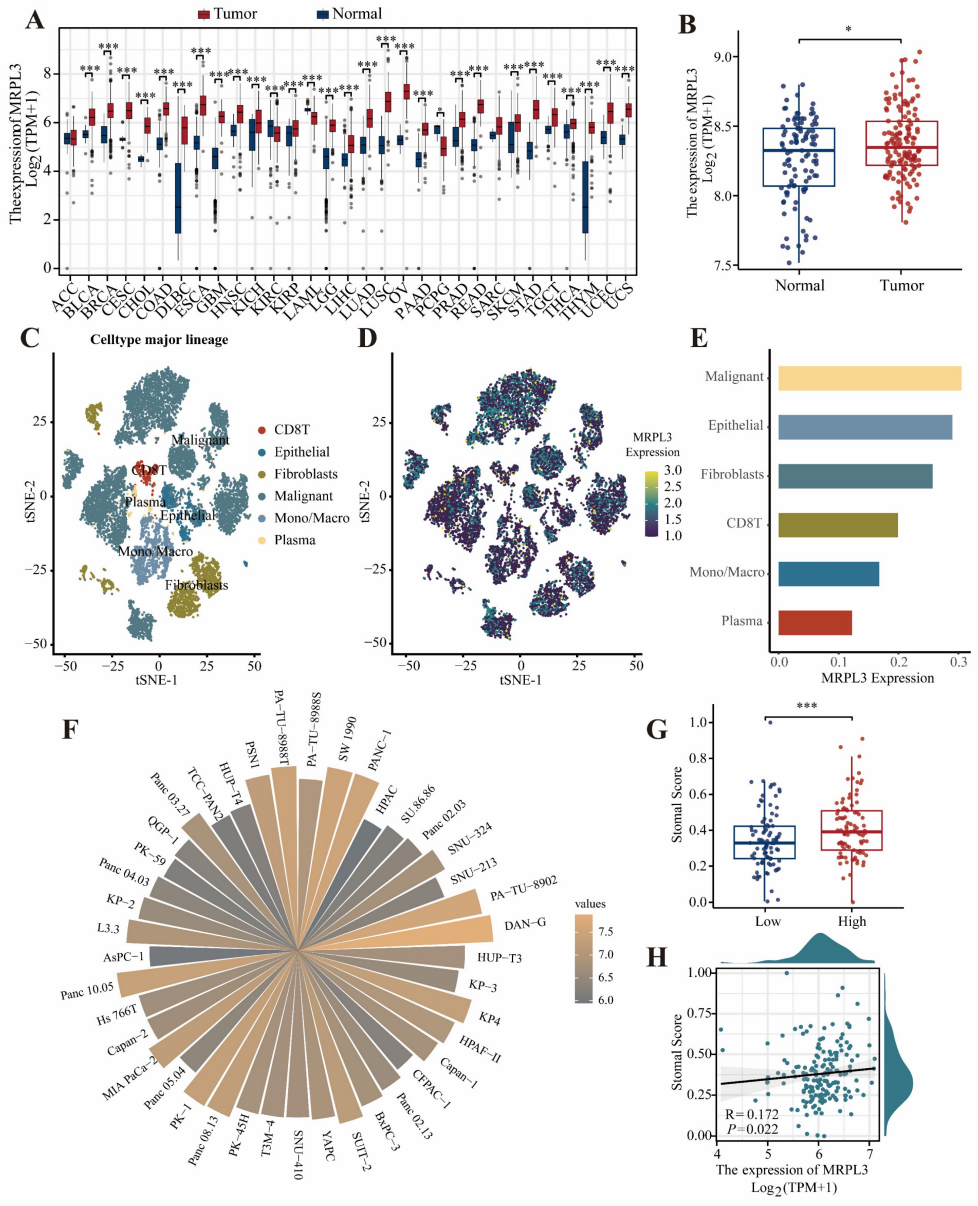
** MRPL3 expression across cancer types and PC. (A)** Pan-cancer analysis of MRPL3 expression in TCGA and GTEx datasets. **(B)** A comparison of MRPL3 expression between PC tissues and normal pancreatic tissues in the GSE183795 dataset. **(C)** t-SNE plot showing single-cell clustering. **(D)** t-SNE plot illustrating MRPL3 expression distribution. **(E)** Bar graph showing MRPL3 expression abundance across different cell types. **(F)** Circular bar plot depicting MRPL3 expression in different PC cell lines (CCLE). **(G)** Box plot of stemness scores comparing MRPL3-high and MRPL3-low groups. **(H)** Scatter plot between stemness scores and MRPL3 expression.

Pan-cancer analysis of TCGA and GTEx datasets revealed significantly elevated MRPL3 expression across different cancer types, including BLCA, BRCA, CESC, CHOL, COAD, DLBC, and PAAD, compared to that in normal tissues (Figure 1A). GSE183795 analysis further confirmed the marked MRPL3 upregulation in PC tissues (Figure 1B). Single-cell RNA-seq analysis (GSE154778) revealed that MRPL3 was markedly elevated in tumor cells compared to endothelial and fibroblast cells (Figure 1C-E). Analysis of the CCLE database revealed diverse MRPL3 expression across 40 PC cell lines (Figure 1F). In addition, patients with PC with high MRPL3 expression exhibited significantly higher tumor stemness scores, supporting the association between MRPL3 overexpression and aggressive tumor phenotypes (Figure 1G-H).

### Prognostic Significance of MRPL3 in PC Patients in TCGA

**Figure 2:**
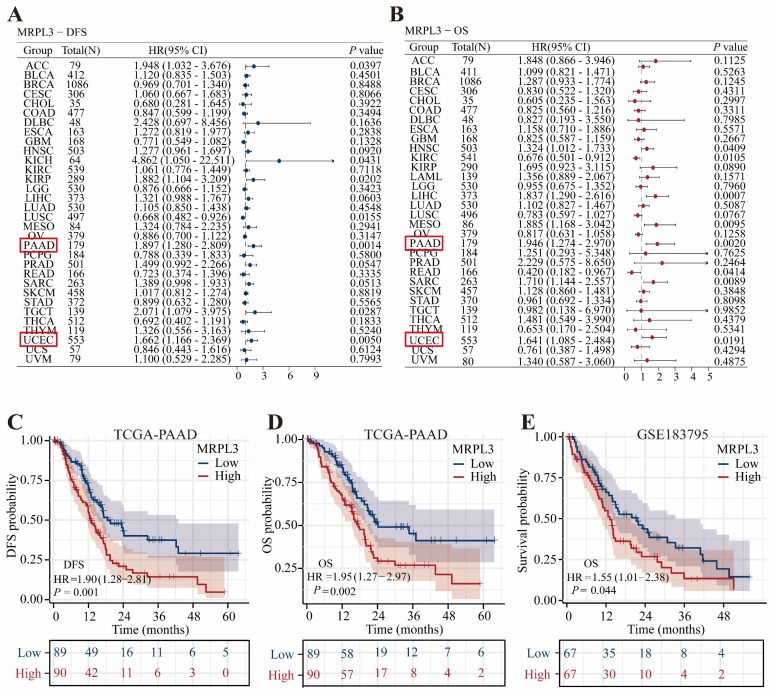
** Prognostic significance of MRPL3 expression. (A-B)** Forest plots of univariate Cox regression analyses of MRPL3 for DFS and OS across cancer types (pan-cancer). **(C-D)** Kaplan-Meier survival curves comparing DFS and OS between MRPL3-high and MRPL3-low groups (TCGA-PAAD dataset). **(E)** Kaplan-Meier survival curve comparing OS in the GSE183795 dataset.

Pan-cancer univariate Cox regression analysis revealed that MRPL3 was a significant prognostic factor for disease-free survival (DFS) and OS, specifically in PC and uterine corpus endometrial carcinoma (Figure 2A-B). Survival analysis demonstrated that high MRPL3 expression was associated with shorter DFS (hazard ratio [HR] = 1.90, 95% confidence interval [CI] = 1.28-2.18, *p* = 0.001) and OS (HR = 1.95, 95% CI = 1.27-2.97, *p* = 0.002) in TCGA-PAAD, as well as with poorer OS (HR = 1.55, 95% CI = 1.01-2.38, *p* = 0.044) in GSE183795 (Figure 2C-E). Further analysis of the clinicopathological features indicated that elevated MRPL3 expression was significantly correlated with advanced T stage and poorer histological grade (*p* < 0.05, Figure S1). In addition, stratified analyses demonstrated shorter OS in patients with high MRPL3 expression across multiple subgroups (Figure S2). Multivariate Cox regression analysis confirmed that MRPL3 expression was an independent predictor of OS in patients with PC (Table S2).

### Identification of DEGs and Functional Enrichment Analysis

**Figure 3:**
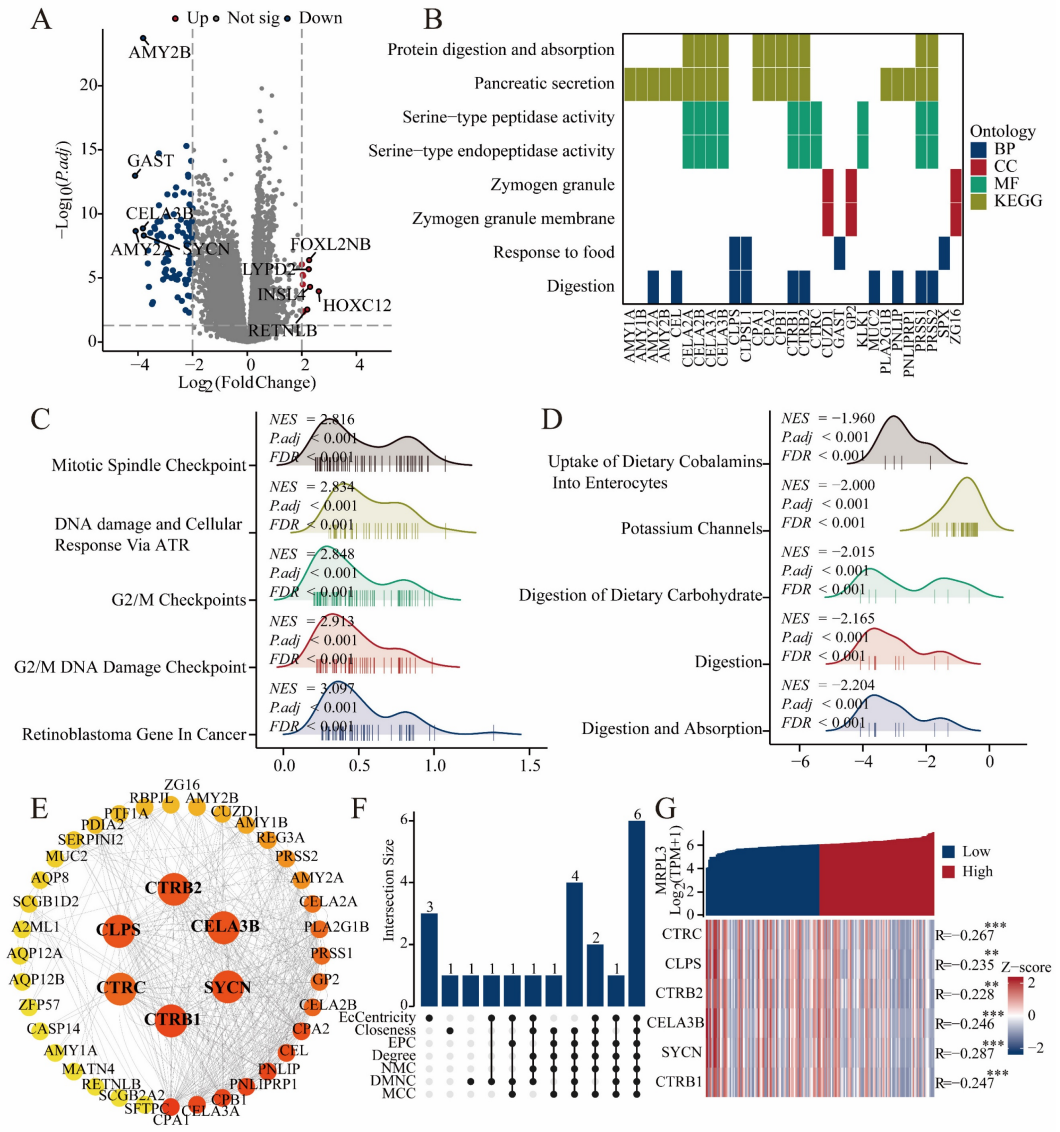
** DEGs, enrichment analysis, and PPI network. (A)** Volcano plot showing DEGs between MRPL3-high and MRPL3-low groups. **(B)** GO and KEGG pathway enrichment analyses of DEGs. **(C-D)** GSEA results showing enriched pathways associated with the MRPL3-high versus MRPL3-low groups. **(E)** Protein-protein interaction (PPI) network of MRPL3-associated genes. **(F)** Hub genes selected by intersection analysis. **(G)** Heatmap illustrating the correlation between MRPL3 and the hub genes.

In total, 182 DEGs (22 upregulated and 160 downregulated) were identified between the high and low MRPL3 expression groups. The top five up-regulated *DEGs* were *FOXL2NB*, *LYPD2*, *INSL4*, *HOXC12*, and *RETNLB*, whereas the top five down-regulated DEGs were *AMY2B, GAST, CELA3B, SYCN*, and *AMY2A* (Figure 3A). The GO analysis suggested that these DEGs were primarily involved in digestive processes and responses to food (biological processes), zymogen granule-related functions (cellular components), and serine-type peptidase activity (molecular functions). The KEGG enrichment highlighted pathways in pancreatic secretion, protein digestion, and absorption (Figure 3B; Table S3). GSEA revealed that MRPL3 upregulation was associated with the G2/M damage checkpoint and mitotic spindle checkpoint pathways, whereas MRPL3 downregulation was linked to the digestion and absorption pathways (Figure 3C-D).

### PPI Network Analysis

A PPI network of MRPL3-associated genes was constructed using STRING and Cytoscape to identify six hub genes (*CLPS, CTRB2, CELA3B, SYCN, CTRB1*, and *CTRC*) through the intersection of top candidates from multiple algorithms. These genes demonstrated significant negative correlations with MRPL3 expression, as visualized by heat maps (Figure 3E-G).

### Immune Cell Infiltration Analysis

**Figure 4:**
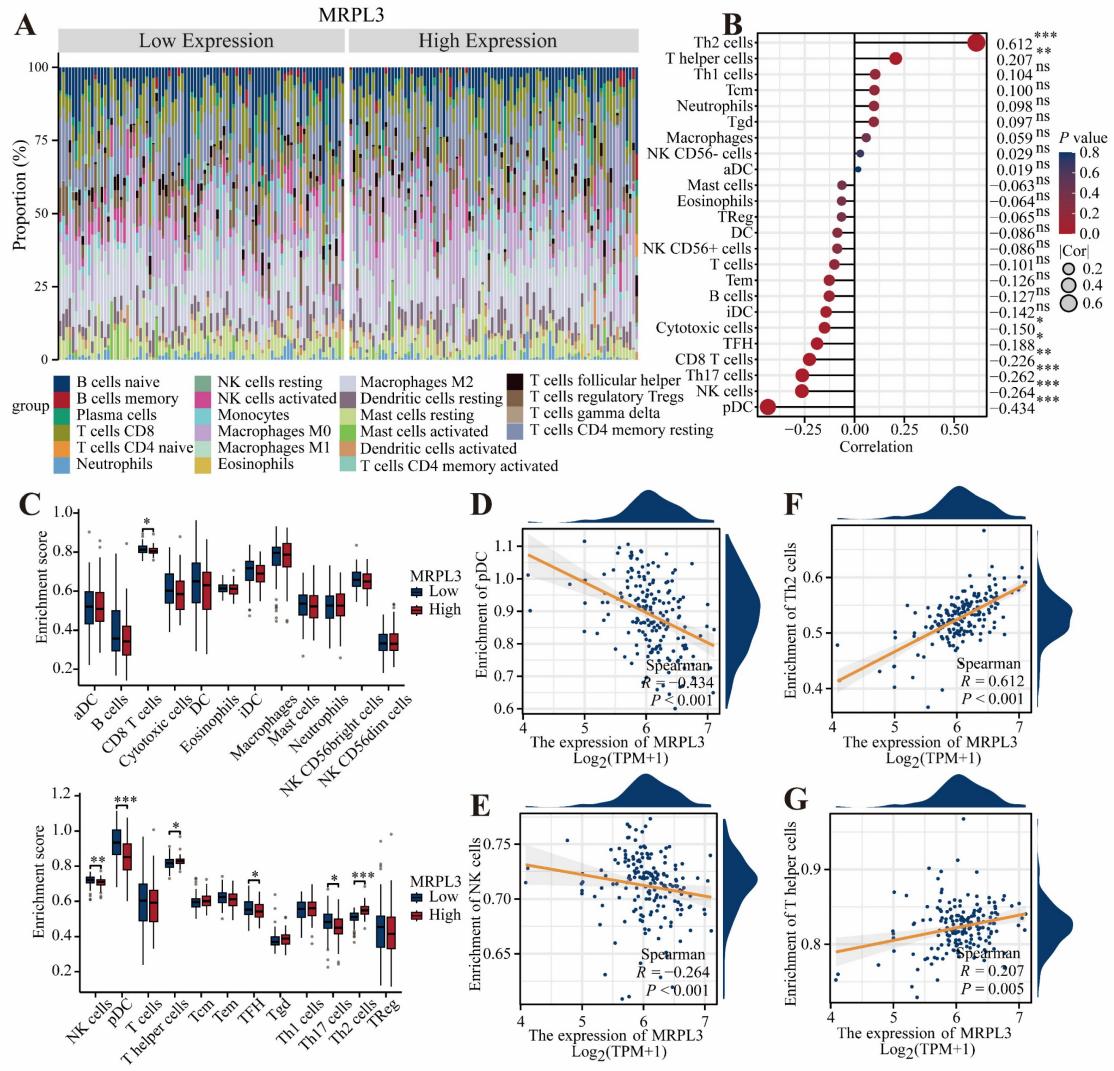
** Association of MRPL3 with immune infiltration in PC. (A)** Bar chart showing the proportions of 22 immune cell types in PC samples. **(B)** Bubble plot illustrating correlation strength between MRPL3 expression and immune cell infiltration. **(C)** Immune infiltration levels were compared between the MRPL3-high and MRPL3-low groups. **(D-G)** Scatter plots depicting correlations of MRPL3 expression with infiltration levels of pDC, NK cells, Th2 cells, and T helper cells.

The CIBERSORT analysis demonstrated that elevated MRPL3 expression was associated with increased Th2 and T helper cell infiltration; however, decreased infiltration of plasmacytoid dendritic cells (pDC), natural killer (NK) cells, Th1 cells, and CD8 + T cells (Figure 4A-C). Scatter plots further confirmed the relationship between MRPL3 expression and immune cell numbers (Figure 4D-G).

### MRPL3 Genetic Variation in PC

**Figure 5:**
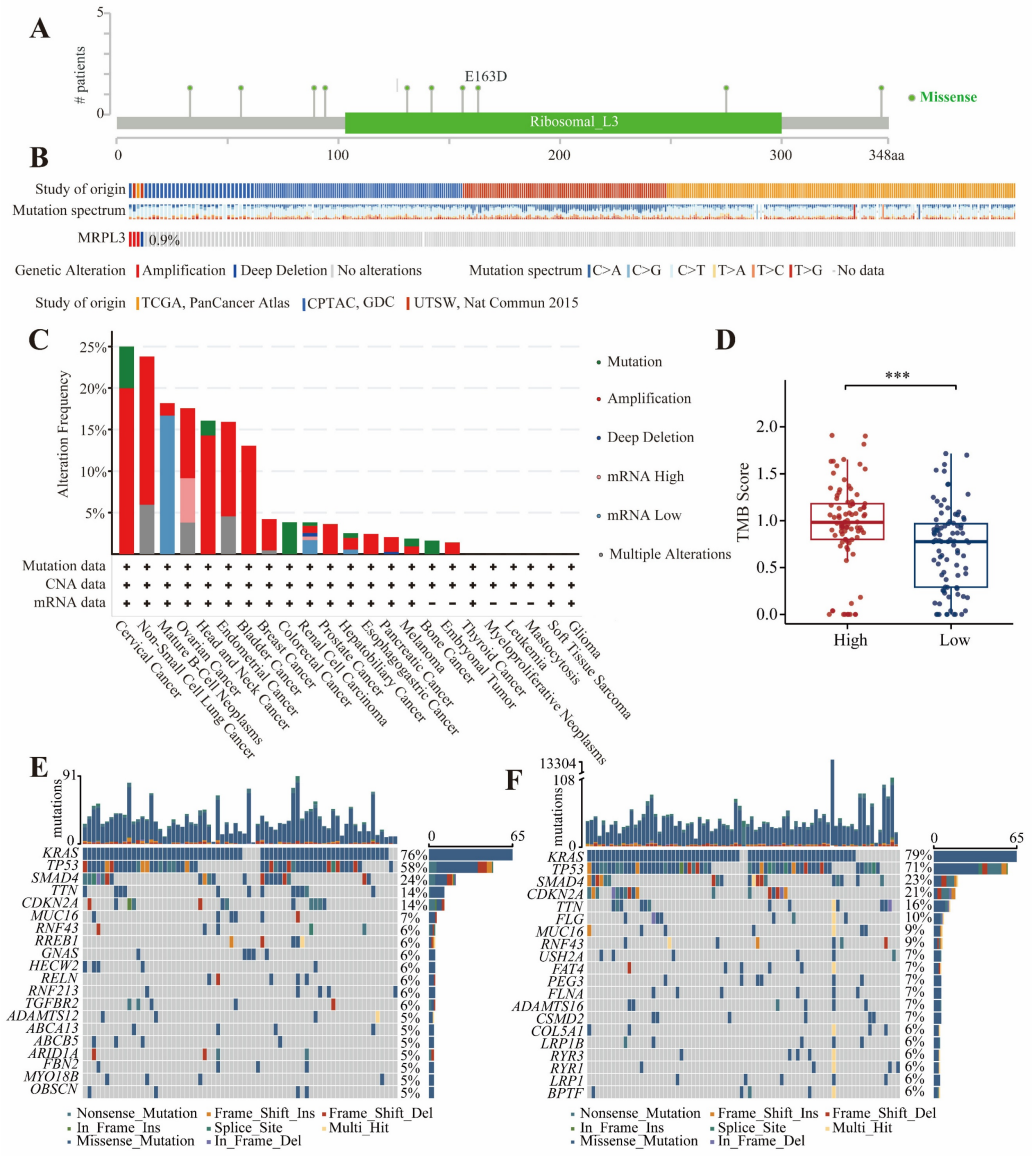
** Mutations levels of* MRPL3* in PC. (A)** Distribution of *MRPL3* mutations across protein domains. **(B-C)** Frequency and types of *MRPL3* gene alterations (cBioPortal pan-cancer analysis). **(D)** Comparison of tumor mutation burden (TMB) between MRPL3-high and MRPL3-low groups in PC. **(E-F)** Top 20 frequently mutated genes in MRPL3-low and MRPL3-high groups.

The genomic data from cBioPortal indicated that MRPL3 alterations were the most frequent in cervical cancer (25%). In PC, the frequency of MRPL3 amplification or deep deletions was approximately 0.9% (Figure 5A-C). *KRAS* and *TP53* mutations were prevalent in both the MRPL3 high and low-expression groups (Figure 5E-F). The TMB analysis revealed significantly higher TMB scores in the high MRPL3 group (*p* < 0.001, Figure 5D).

### Immunotherapy Response, Drug Sensitivity, and Therapeutic Compound Screening

**Figure 6:**
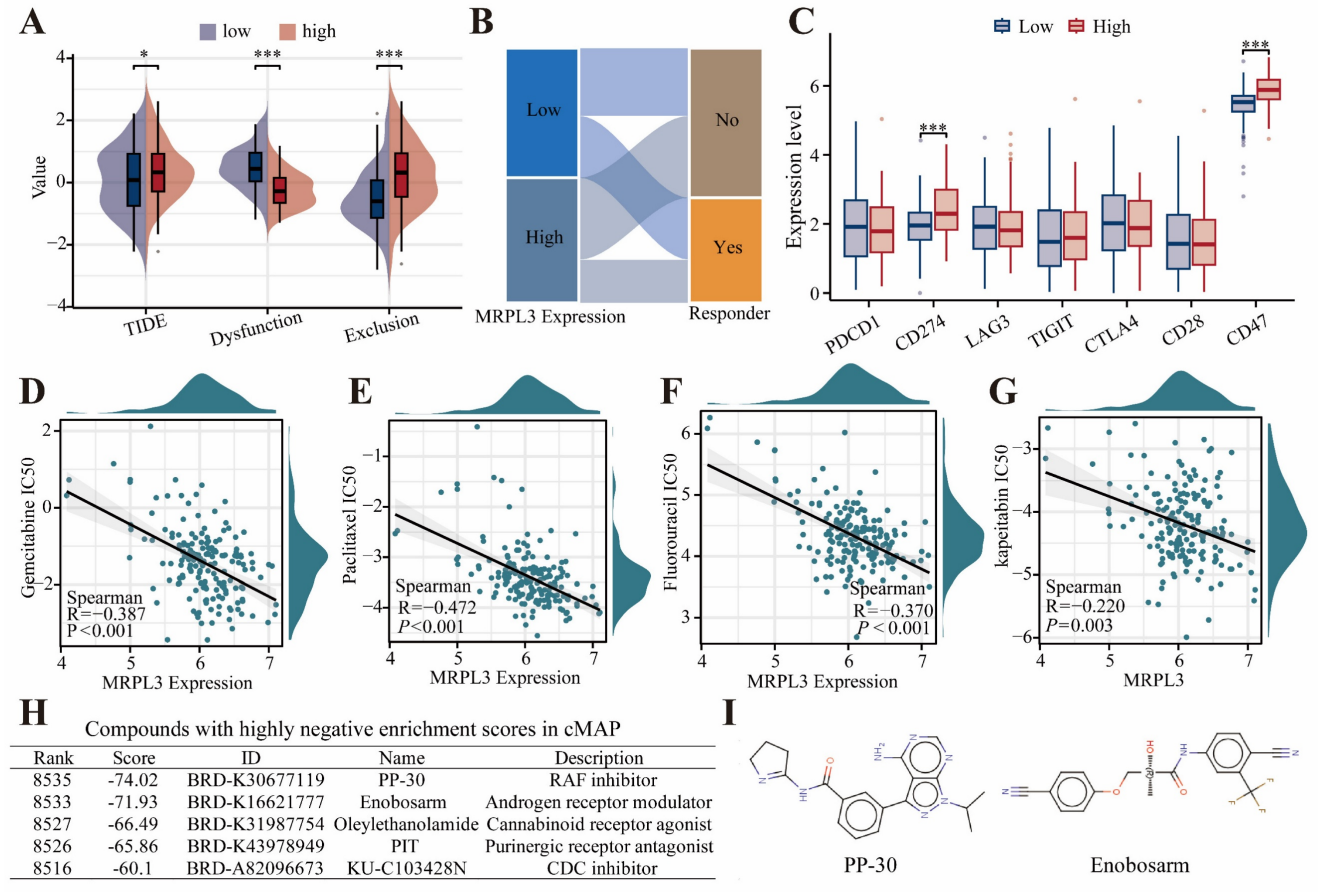
** TIDE and drug Sensitivity analyses in PC. (A)** Immunotherapy response prediction using TIDE in two groups. **(B)** Sankey diagram showing immunotherapy response distribution predicted by TIDE. **(C)** Expression of immune checkpoint genes was compared between the MRPL3-high and MRPL3-low groups. **(D-G)** Drug sensitivity (IC_50_ values) analyses of chemotherapeutic agents (gemcitabine, paclitaxel, fluorouracil, and capecitabine) relative to MRPL3 expression. **(H)** List of potential therapeutic compounds identified by cMap analysis (top five lowest enrichment scores). **(I)** Chemical structures of PP-30 and enobosarm.

Patients with high MRPL3 expression had higher TIDE scores, indicating lower predicted immunotherapy response rates (Figure 6A-B). High MRPL3 expression also correlated with increased expression of immune checkpoint genes, such as* CD274* and *CD47* (Figure 6C). Drug sensitivity analysis demonstrated that increased MRPL3 expression was associated with significantly decreased IC_50_ values for gemcitabine, paclitaxel, fluorouracil, and capecitabine (*p* < 0.05; Figure 6D-G). The cMap analysis identified five compounds, particularly RAF kinase inhibitors, and androgen receptor modulators, as potential therapeutic agents for the MRPL3-high PC group (Figure 6H, I).

### Clinical Validation of MRPL3 via IHC

**Figure 7:**
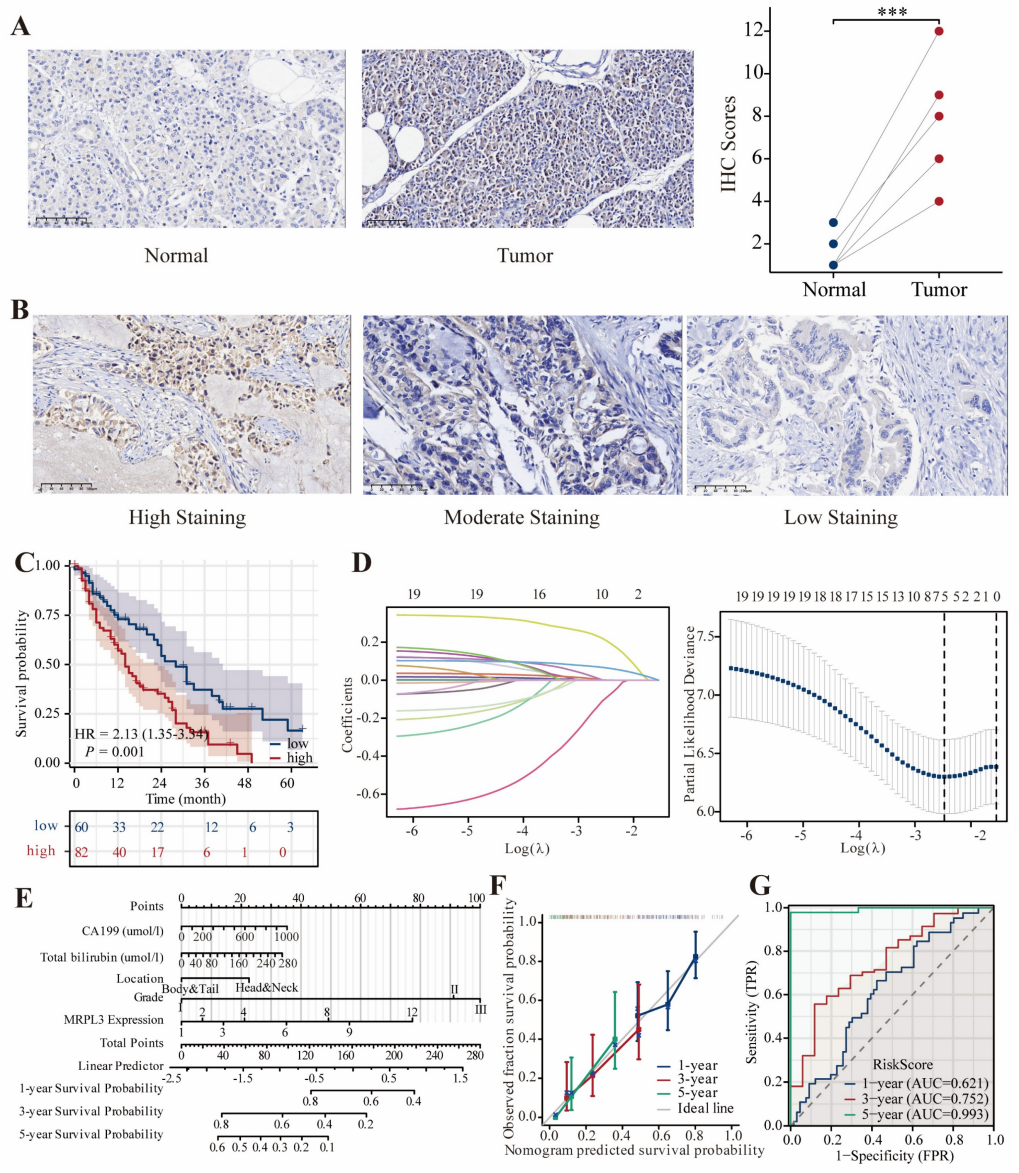
** MRPL3 expression validation in a clinical cohort. (A)** Representative IHC images of MRPL3 expression in PC and normal tissues (×200), confirmed that MRPL3 expression was significantly higher in tumor tissue. **(B)** Representative IHC images of MRPL3 expression in PC tissues (×200). **(C)** Kaplan-Meier survival curves comparing OS between the two groups. **(D-E)** Prognostic features identified via LASSO regression and lambda value plot. **(F-G)** Nomogram for OS prediction and corresponding calibration and ROC curves to validate nomogram accuracy.

Representative immunohistochemical images are shown in Figure 7A-B. IHC analysis of six paired PC samples revealed significantly higher MRPL3 expression in tumor tissues than in normal tissues. In a clinical cohort of 142 patients with PC, IHC revealed that high MRPL3 expression was associated with significantly shorter OS (HR = 2.13, 95% CI = 1.35-3.34, *p* = 0.001) (Figure 7C). The multivariate Cox regression analysis identified MRPL3 as an independent prognostic factor (Table 2). LASSO regression identified MRPL3 expression, CA19-9 levels, total bilirubin levels, tumor location, and histological grade as prognostic indicators of OS (Figure 7D-E). A prognostic nomogram incorporating these factors demonstrated strong predictive accuracy, with areas under the curves of 0.621, 0.752, and 0.993 at 1, 3, and 5 years, respectively (Figure 7F-G).

### *In vitro* Functional Validation of MRPL3

**Figure 8:**
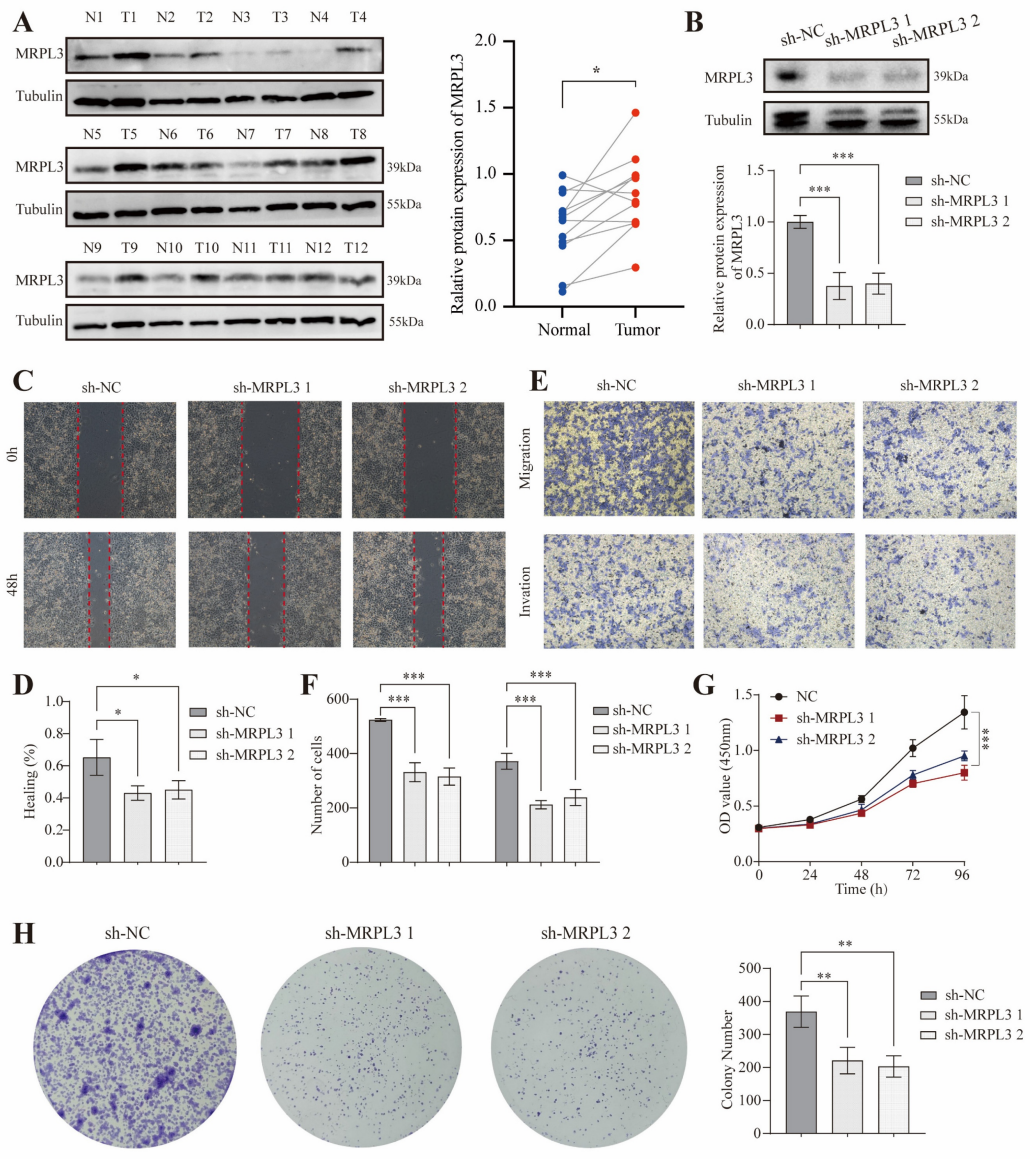
***In vitro* cell experiment of MRPL3 in PC. (A)** Western blot confirming MRPL3 protein levels in paired PC and normal pancreatic tissues. **(B)** Western blot validation of *MRPL3* knockdown in SW1990 cells. **(C-D)** Wound healing assays demonstrate reduced migration capacity after *MRPL3* knockdown. **(E-F)** Transwell migration and invasion assays demonstrating suppressed cell migration and invasion after* MRPL3* knockdown. **(G)** CCK-8 proliferation assay showing decreased proliferation after *MRPL3* knockdown. **(H)** Colony formation assay confirming impaired clonogenic ability following *MRPL3* knockdown in SW1990 cells. PC: pancreatic cancer. ns, *p* ≥ 0.05; *, *p* < 0.05; **, *p* < 0.01; ***,* p* < 0.001

Western blot analysis of paired tumor and adjacent normal pancreatic tissues from 12 patients confirmed significantly elevated MRPL3 protein levels in PC tissues (*p* < 0.001; Figure 8A). To evaluate MRPL3's function, we established MRPL3-knockdown PC cell lines as confirmed by western blotting (Figure 8B). Wound healing and Transwell assays demonstrated that* MRPL3* knockdown significantly reduced PC cell migration and invasion (Figure 8C-F). The CCK-8 assay revealed that* MRPL3* knockdown markedly inhibited SW1990 cell proliferation (Figure 8G). In addition, colony formation assays confirmed that the *MRPL3* knockdown substantially impaired the clonogenic capacity of PC cells (Figure 8H).

## Discussion

The inherent complexity, aggressive invasiveness, and early metastatic potential of PC pose significant challenges to its clinical management. Consequently, even after curative surgical resection, the 5-year survival rate remains below 10% 16. Consistent with previous reports, our cohort had a median survival of 1.58 years and a 5-year survival rate of 10.8%. Thus, the identification of novel therapeutic targets and molecular biomarkers is critical for improving the outcomes of patients with PC.

MRPL3 is a structural component of mitochondrial ribosomes. Previous studies suggested that MRPL3 regulates lactylation and metabolic reprogramming 12. Elevated MRPL3 expression has been linked to poor prognosis in prostate cancer and hepatocellular carcinoma, correlating with advanced tumor stage and invasiveness 12,17. In addition, models incorporating MRPL3 have demonstrated strong prognostic performance in lung and breast cancers 9,18. Here, we demonstrated that MRPL3 expression is significantly elevated in PC tissues and is associated with poor survival outcomes. IHC analysis of 142 patients with resected PC confirmed that high MRPL3 expression corresponded to significantly reduced OS, and multivariate analysis established MRPL3 as an independent prognostic factor. Western blotting and IHC validation further confirmed that MRPL3 was upregulated in PC. Interestingly, the Cox regression analysis indicated that TNM staging was not an independent prognostic factor for PC. This could be attributed to several factors. First, tumor size and lymph node metastasis were included in the regression analysis, both of which are key components of the TNM staging system. These variables may have influenced the results of the Cox regression analysis. Second, the relatively small sample size of this study may have provided insufficient power to detect the prognostic effect of TNM staging, potentially introducing bias. Third, TNM staging depends primarily on morphological features, which may not comprehensively reflect the molecular characteristics of the disease. Consequently, TNM staging alone may not provide a sufficient basis for stratifying the prognosis of patients with PC.

Cancer stemness refers to the properties of a subset of cancer cells, cancer stem cells (CSCs), which can switch between differentiated and stem-like states, driving tumor initiation, self-renewal, and differentiation 19. Generally, higher stemness is associated with greater invasion, metastasis, and resistance to therapy 20. Consistent with this concept, we observed significantly higher stemness scores in patients with high MRPL3 expression, suggesting that MRPL3 may indicate a more aggressive tumor phenotype and serve as a prognostic marker for PC.

*In vitro* experiments demonstrated that *MRPL3* knockdown markedly inhibited PC cell proliferation, migration, and invasion, suggesting that MRPL3 is a potential therapeutic target. These findings suggest promising directions for the development of future PC therapies.

The G2/M checkpoint is crucial for maintaining genomic stability by preventing cells with damaged DNA from entering mitosis 21,22. If this checkpoint is impaired, cells can proceed through division with unrepaired DNA, leading to genomic instability and tumor progression 23. Furthermore, defective G2/M checkpoints may contribute to immune evasion 24. Our enrichment analysis indicated that high MRPL3 expression was significantly associated with pathways involving the mitotic spindle checkpoint, G2/M DNA damage checkpoint, DNA damage, and cellular response via ATR, suggesting that MRPL3 may promote PC progression by influencing G2/M checkpoint control.

The TME significantly influences PC progression. Our immune analysis demonstrated that elevated MRPL3 expression correlated with increased infiltration of Th2 and T helper cells, whereas reduced MRPL3 expression was associated with higher levels of CD8 + T, NK, and Th1 cells. The Th1/Th2 balance is maintained under normal physiology; however, in cancer, elevated Th2 cytokines skew this balance toward a Th2-dominant response 25. Th2 cells produce interleukin (IL)-4 and IL-13, which inhibit cytotoxic T-cell function and diminish anti-tumor immune responses 26. Conversely, CD8 + T and NK cells directly kill tumor cells or enhance adaptive immunity; however, in PC, these cells are often exhausted by the immunosuppressive environment. Thus, increased Th2 infiltration associated with high MRPL3 expression may suppress CD8 + T and NK cell activity, thereby fostering an immunosuppressive environment that promotes tumor progression 27-29. Consistent with this, the TIDE analysis demonstrated greater immune exclusion and lower immunotherapy response in patients with high MRPL3 expression, highlighting the potential MRPL3 as a biomarker for immunotherapy outcomes.

Genomic instability driven by key mutations plays a significant role in cancer 30. TMB, which reflects the load of somatic mutations, is an important biomarker for immunotherapy 31,32. TMB expression was significantly higher in patients with PC with high MRPL3 expression. In addition, this group exhibits a high prevalence of *KRAS* and *TP53* mutations. *KRAS* mutations, which are present in approximately 90% of PC cases, drive tumor progression through constitutive MAPK/ERK and PI3K/AKT signaling, influencing the TME and promoting tumor stemness 33,34. *TP53* mutations, observed in 65% of cases, are associated with poor OS 35. Despite the prevalence of oncogenic mutations, targeted therapies have achieved limited success and necessitate further research 33.

Interestingly, we found that high MRPL3 expression was associated with increased sensitivity to chemotherapeutic agents such as gemcitabine, paclitaxel, and fluorouracil. Gemcitabine inhibits DNA synthesis, paclitaxel disrupts microtubules, and fluorouracil impairs DNA synthesis and repair, thereby improving outcomes in advanced PC, especially in combination regimens (e.g., gemcitabine plus paclitaxel or FOLFIRINOX) 36-38. The high MRPL3 expression observed in this study enhanced chemotherapy sensitivity in PC, possibly because of the role of MRPL3 in ribosome function. Platinum-based drugs may induce ribosome biogenesis to kill tumor cells rather than directly causing DNA damage 39. Ribosome synthesis is crucial in cancer, and its disruption can significantly affect the efficacy of chemotherapy 40. Thus, MRPL3 may be a predictive biomarker for chemotherapy response and a target for overcoming chemoresistance. Raf kinase is a key enzyme in the MAPK/ERK pathway and is aberrantly activated in cancer cells. Raf inhibitors disrupt this pathway, inhibit tumor cell proliferation and invasion, and act synergistically with other treatments 41,42. Our cMap analysis identified Raf kinase inhibitors as promising candidates for treating patients with PC with high MRPL3 expression. These findings may guide the development of novel targeted therapies for PC.

This study has several limitations. First, our conclusions were based primarily on bioinformatic analyses, clinical cohort data, and *in vitro* experiments; thus, the precise mechanisms by which MRPL3 influences PC and its specific pathways remain unclear. Second, although validation was conducted using publicly accessible databases and our clinical cohort, the sample size was relatively small and included patients from a single institution. In addition, although our predictive model demonstrated strong predictive efficacy, its application value was confirmed through a single clinical cohort validation; however, external validation datasets are currently lacking to further assess the model's stability. Larger multicenter studies are required to confirm the clinical significance of MRPL3 expression.

## Conclusions

In conclusion, our findings indicate that elevated MRPL3 expression correlates with poor prognosis in PC and immunosuppressive TME, marked by increased Th2 cell infiltration and G2/M checkpoint dysregulation. Functional validation confirmed that MRPL3 silencing suppresses the malignant phenotype of PC cells. These findings establish MRPL3 as a promising prognostic biomarker and therapeutic target, providing insights into precision medicine and the prognostic stratification of PC.

## Supplementary Material

Supplementary figures and tables.

## Figures and Tables

**Table 1:** Relationship between MRPL3 expression and clinicopathological features in clinical cohort.

Characteristics	High expression (82)	Low expression (60)	*P* value
Gender, n (%)			0.803
Female	40 (48.8%)	28 (46.7%)	
Male	42 (51.2%)	32 (53.3%)	
Age (years), M(IQR)	62 (56, 67)	62 (57, 69)	0.389
Diabetes history, n (%)			0.924
Yes	10 (12.2%)	7 (11.7%)	
No	72 (87.8%)	53 (88.3%)	
Smoking history, n (%)			0.992
No	52 (63.4%)	38 (63.3%)	
Yes	30 (36.6%)	22 (36.7%)	
weight loss (kg), n (%)			0.105
No	61 (74.4%)	37 (61.7%)	
Yes	21 (25.6%)	23 (38.3%)	
CA199 (nmol/L), M (IQR)	156.88 (31.06, 454.65)	137.5 (38.395, 396.1)	0.398
CEA (ng/ml), M (IQR)	3.54 (1.9125, 6.5925)	4.02 (2.5075, 5.835)	0.404
Albumin(g/L), M (IQR)	44.65 (41.3, 45.8)	44.8 (41.725, 46.1)	0.541
Total bilirubin (umol/l), M (IQR)	15.9 (10.4, 105.7)	13.5 (7.8, 48.925)	0.037
NLR, M (IQR)	2.9074 (2.1826, 3.8104)	2.7153 (2.0208, 3.6816)	0.408
PLR, M (IQR)	216.86 (182.65, 256.14)	235.92 (203.4, 267.35)	0.035
Location, n (%)			0.801
Head and Neck	53 (64.6%)	40 (66.7%)	
Body and Tail	29 (35.4%)	20 (33.3%)	
Size (cm), M (IQR)	3.5 (2.525, 4.5)	3.2 (2.7, 4.525)	0.699
Lymph node involvement, n (%)			0.933
Yes	32 (39%)	23 (38.3%)	
No	50 (61%)	37 (61.7%)	
Perineural invasion, n (%)			0.498
No	23 (28%)	20 (33.3%)	
Yes	59 (72%)	40 (66.7%)	
MVI, n (%)			0.148
No	66 (80.5%)	42 (70%)	
Yes	16 (19.5%)	18 (30%)	
Grade, n (%)			0.310
I	6 (7.3%)	9 (15%)	
II	54 (65.9%)	38 (63.3%)	
III	22 (26.8%)	13 (21.7%)	
Postoperative chemotherapy, n (%)			0.360
No	46 (56.1%)	29 (48.3%)	
Yes	36 (43.9%)	31 (51.7%)	
Postoperative complications, n (%)			0.281
No	67 (81.7%)	53 (88.3%)	
Yes	15 (18.3%)	7 (11.7%)	
TNM stage, n(%)			0.890
I	37 (45.1%)	29 (48.3%)	
II	35 (42.7%)	25 (41.7%)	
III	10 (12.1%)	6 (10.0%)	
MRPL3 Expression	9 (8,12)	4 (3,6)	<0.001

M (IQR): Median (Interquartile Range). CA199: Carbohydrate Antigen 199, CEA: Carcinoembryonic antigen. NLR: Neutrophil-to-Lymphocyte Ratio. PLR: Platelet-to-Lymphocyte Ratio. MVI: Microvascular Invasion.

**Table 2:** Univariate and multivariate Cox regression analysis for OS in clinical cohort.

Characteristics	Total (N)	Univariate analysis			Multivariate analysis	
		Hazard ratio (95% CI)	P value		Hazard ratio (95% CI)	P value
Gender	142					
Female	68	Reference				
Male	74	1.336 (0.872 - 2.047)	0.183			
Age (years)	142	1.010 (0.988 - 1.032)	0.387			
Diabetes history	142					
Yes	17	Reference				
No	125	0.872 (0.474 - 1.604)	0.660			
Smoking history	142					
No	90	Reference				
Yes	52	1.265 (0.822 - 1.948)	0.285			
weight loss (kg)	142					
No	98	Reference				
Yes	44	0.883 (0.559 - 1.395)	0.595			
CA199 (nmol/L)	142	1.001 (1.000 - 1.001)	0.074		1.001 (1.000 - 1.001)	0.057
CEA (ng/ml)	142	1.006 (0.990 - 1.023)	0.435			
Albumin(g/L), M (IQR)	142	0.972 (0.911 - 1.037)	0.394			
Total bilirubin (umol/l)	142	1.003 (1.000 - 1.006)	0.060		1.002 (0.999 - 1.005)	0.256
NLR	142	1.034 (0.994 - 1.074)	0.093		1.031 (0.991 - 1.074)	0.132
PLR	142	1.001 (0.996 - 1.006)	0.664			
Location	142					
Head and Neck	93	Reference			Reference	
Body and Tail	49	0.656 (0.415 - 1.037)	0.071		0.702 (0.432 - 1.142)	0.154
Size (cm)	142	1.078 (0.962 - 1.208)	0.195			
Lymph node involvement	142					
Yes	55	Reference				
No	87	0.893 (0.586 - 1.360)	0.597			
Perineural invasion	142					
No	43	Reference				
Yes	99	0.934 (0.597 - 1.460)	0.765			
MVI	142					
No	108	Reference				
Yes	34	1.095 (0.679 - 1.765)	0.710			
Grade	142					
I	15	Reference			Reference	
II	92	4.079 (1.468 - 11.333)	**0.007**		3.824 (1.367 - 10.695)	**0.011**
III	35	4.431 (1.533 - 12.813)	**0.006**		4.262 (1.456 - 12.473)	**0.008**
Postoperative chemotherapy	142					
No	75	Reference				
Yes	67	0.860 (0.568 - 1.301)	0.474			
Postoperative complications	142					
No	120	Reference				
Yes	22	1.144 (0.655 - 1.998)	0.636			
TNM Stage						
I	66	Reference				
II	60	1.089 (0.703 - 1.686)	0.701			
III	16	1.058 (0.524 - 2.132)	0.875			
MRPL3 expression	142	1.108 (1.039 - 1.181)	**0.002**		1.102 (1.031 - 1.178)	**0.004**

OS: Overall survival. CA199: Carbohydrate Antigen 199, CEA: Carcinoembryonic antigen. NLR: Neutrophil-to-Lymphocyte Ratio. PLR: Platelet-to-Lymphocyte Ratio. MVI: Microvascular Invasion.
