# Supplementary material for: An integrated analysis identified mitochondrial ribosomal protein MRPL3 as a potential prognostic biomarker and therapeutic target in pancreatic cancer
Source: J Cancer. 2025 Jul 11;16(10):3244–60. doi: 10.7150/jca.114067 (PMC12305564; doi:10.7150/jca.114067)
Supplement: Supplementary file 1 — Supplementary figures and tables. [file jcav16p3244s1.pdf]

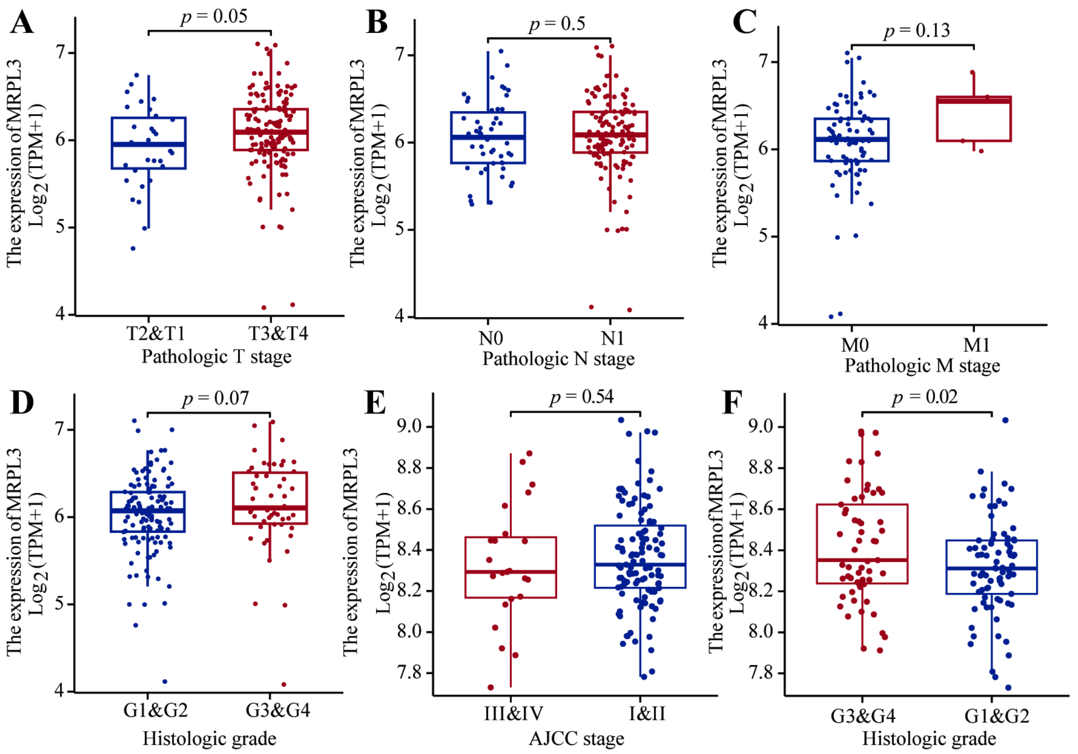

Figure S1. Association between MRPL3 expression and clinicopathological characteristics. Analysis based on the TCGA dataset: (A) T stage, (B) N stage, (C) M stage, (D) histologic grade, and (E) pathological stage. Analysis based on the GSE183795 dataset: (F) histologic grade. TCGA, The Cancer Genome Atlas.

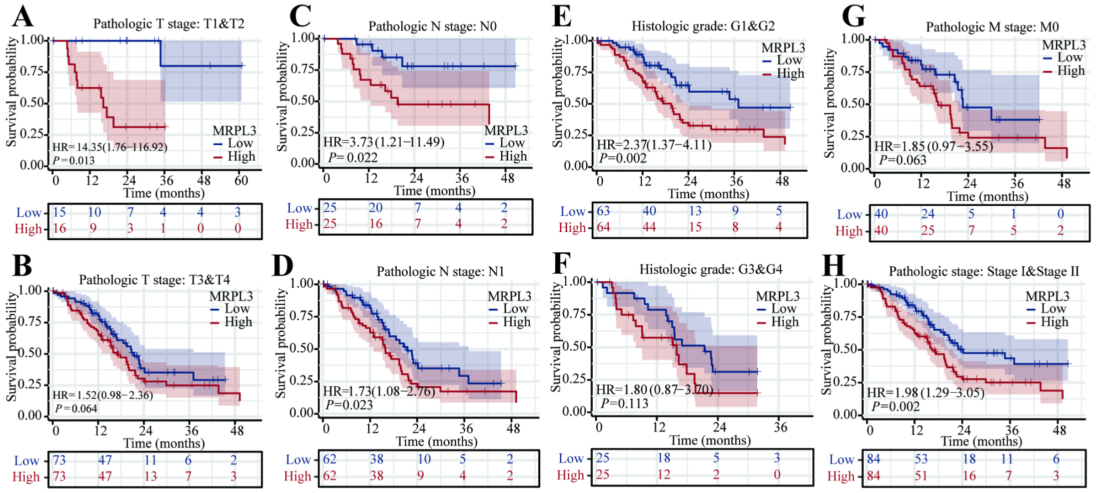

Figure S2. Kaplan–Meier survival analysis for OS stratified by clinicopathological subgroups. Kaplan–Meier survival curves for OS based on TCGA dataset: (A) T1–T2 stage patients, (B) T3–T4 stage patients, (C) N0 stage patients, (D) N1 stage patients, (E) G1–G2 histologic grade patients, (F) G3–G4 histologic grade patients, (G) M0 stage patients, and (H) Stage I–II patients.

OS, overall survival.

Table S1. The relationship between MRPL3 expression and clinicopathological features in TCGA

Characteristics (n)	Low expression (89)	High expression (90)	*P* value
Gender, n (%)			0.713
Female	41 (46.1%)	39 (43.3%)	
Male	48 (53.9%)	51 (56.7%)	
Age, years, n (%)			0.498
≤65	49 (55.1%)	45 (50%)	
> 65	40 (44.9%)	45 (50%)	
Pathologic stage, n (%)			0.183
Stage I	11 (12.4%)	10 (11.1%)	
Stage II	73 (82.0%)	74 (82.2%)	
Stage III	3 (3.4%)	0 (0%)	
Stage IV	1 (1.1%)	4 (4.5%)	
Unknown	1 (1.1%)	2 (2.2%)	
Histologic grade, n (%)			0.345
G1	20 (22.5%)	11 (12.2%)	
G2	45 (50.6%)	51 (56.7%)	
G3	22 (24.7%)	26 (28.9%)	
G4	1 (1.1%)	1 (1.1%)	
Unknown	1 (1.1%)	1 (1.1%)	
Alcohol history, n (%)			0.392
No	30 (33.7%)	35 (38.9%)	
Yes	54 (60.7%)	48 (53.3%)	
Unknown	5 (5.6%)	7 (7.8%)	
History of diabetes, n (%)			0.243
No	54 (60.7%)	55 (61.1%)	
Yes	23 (25.8%)	15 (16.7%)	

Characteristics (n)	Low expression (89)	High expression (90)	*P* value
Unknown	12 (13.5%)	20 (22.2%)	
History of pancreatitis, n (%)			0.652
No	68 (76.4%)	61 (67.8%)	
Yes	6 (6.7%)	7 (7.8%)	
Unknown	15 (16.9%)	22 (24.4%)	

Table S2. Univariate and multivariate Cox regression of MRPL3 expression in TCGA

Characteristics	Total(N)	Univariate analysis		Multivariate analysis	
		Hazard ratio (95% CI)	*P* value	Hazard ratio (95% CI)	*P* value
Gender	179				
Female	80	Reference			
Male	99	0.813 (0.541 - 1.222)	0.319		
Age (years)	179				
<= 65	94	Reference			
> 65	85	1.285 (0.853 - 1.937)	0.230		
MRPL3 expression	179	3.069 (1.841 - 5.117)	**< 0.001**	2.651 (1.517 - 4.631)	**< 0.001**
Pathologic T stage	177				
T1&T2	31	Reference		Reference	
T3&T4	146	2.035 (1.079 - 3.838)	**0.028**	1.107 (0.547 - 2.239)	0.777
Pathologic N stage	174				
N0	50	Reference		Reference	
N1	124	2.161 (1.287 - 3.627)	**0.004**	2.062 (1.176 - 3.616)	**0.012**
Pathologic stage	176				
I& II	168	Reference			
III& IV	8	0.676 (0.213 - 2.145)	0.507		
Histologic grade	177				
G1&G2	127	Reference		Reference	
G3&G4	50	1.532 (0.993 - 2.363)	0.054	1.354 (0.871 - 2.103)	0.178
Alcohol history	167				
No	65	Reference			

Characteristics	Total(N)	Univariate analysis		Multivariate analysis	
		Hazard ratio (95% CI)	*P* value	Hazard ratio (95% CI)	*P* value
Yes	102	1.146 (0.739 - 1.779)	0.543		
History of diabetes	147				
No	109	Reference			
Yes	38	0.923 (0.531 - 1.607)	0.778		
History of pancreatitis	142				
No	129	Reference			
Yes	13	1.169 (0.559 - 2.445)	0.679		

Cox regression analyses were performed only with available data in TCGA database.

Table S3. GO and KEGG analysis results

ONTOLOGY	ID	Description	Gene Ratio	*P* adjust
BP	GO:0007586	digestion	10/76	1.5453E-07
BP	GO:0032094	response to food	4/76	0.00591665
CC	GO:0042589	zymogen granule membrane	3/87	0.00141406
CC	GO:0042588	zymogen granule	3/87	0.00154477
MF	GO:0004252	serine-type endopeptidase activity	10/80	5.0464E-07
MF	GO:0008236	serine-type peptidase activity	10/80	5.0464E-07
KEGG	hsa04972	Pancreatic secretion	19/42	2.4742E-24
KEGG	hsa04974	Protein digestion and absorption	11/42	6.8273E-11

Table S4. GSEA results

Description	Set Size	Enrichment Score	NES	*P* adjust
Retinoblastoma gene in cancer	87	0.707	3.097	7.327E-09
G2/M DNA damage checkpoint	93	0.649	2.913	7.327E-09
G2/M checkpoints	166	0.598	2.849	7.327E-09
DNA irdamage and cellular response via ATR	81	0.648	2.834	7.327E-09
Mitotic spindle checkpoint	111	0.616	2.816	7.327E-09
Digestion and absorption	26	-0.829	-2.204	4.222E-07
Digestion	22	-0.843	-2.165	2.362E-06
Digestion of dietary carbohydrate	11	-0.902	-2.015	3.606E-05
Potassium channels	103	-0.606	-2.000	1.229E-06
Uptake of dietary cobalamins into enterocytes	10	-0.893	-1.960	0.0002
